# Explorando o Escore Prognóstico de Nápoles: Uma Chave para Prever o Início da Fibrilação Atrial em Casos de IAMCCST

**DOI:** 10.36660/abc.20250518

**Published:** 2025-07-08

**Authors:** Paulo Magno Martins Dourado

**Affiliations:** 1 Faculdade de Medicina da Universidade de São Paulo São Paulo SP Brasil Faculdade de Medicina da Universidade de São Paulo, São Paulo, SP – Brasil; 2 Clínica Pró-Coração São Paulo SP Brasil Clínica Pró-Coração, São Paulo, SP – Brasil

**Keywords:** Fibrilação atrial, Infarto do Miocárdio com Supradesnível do Segmento ST, Prognóstico

No cenário em constante evolução do tratamento cardíaco, o Escore Prognóstico de Nápoles (Naples Prognostic Score – (NPS)) surgiu como uma ferramenta promissora para prever o início da fibrilação atrial (FA) em pacientes com infarto agudo do miocárdio com supradesnivelamento do segmento ST (IAMCSST).^1^ Este sistema de pontuação inovador oferece insights sobre os desfechos dos pacientes e capacita os profissionais de saúde a tomar decisões informadas em tempo real. Compreender e utilizar este escore pode aprimorar o manejo do paciente e aumentar as taxas de sobrevida. Vamos explorar como o NPS transforma a abordagem para a predição de IAMCSST e FA.

A FA após IAMCSST é uma preocupação crescente na área da medicina cardiovascular.² O cenário pós-IM apresenta inúmeros desafios, com a FA amplificando significativamente o risco de desfechos adversos para os pacientes. Um estudo recente que destaca o NPS traz novas esperanças na busca por prever e tratar complicações de forma eficaz após o infarto do miocárdio.³ Reconhecer o potencial para a detecção precoce de FA de início recente (FANO) — uma condição associada ao aumento da morbidade e mortalidade — melhora a qualidade do atendimento prestado a pacientes com IAMCSST submetidos à intervenção coronária percutânea primária.^4^

O NPS é uma ferramenta abrangente que integra vários biomarcadores indicativos de um estado pró-inflamatório, sendo os principais componentes a relação neutrófilo-linfócito e a relação linfócito-monócito. Esses biomarcadores são cruciais para delinear o estado inflamatório de um paciente e compreender seu estado nutricional — elementos que se tornaram cada vez mais vitais na previsão de desfechos cardiovasculares. A integração desses parâmetros permite que os médicos avaliem o risco de forma mais eficaz, adaptando-o ao perfil fisiológico único de cada paciente.^5^

A importância da FA após IAMCSST não pode ser superestimada. Apesar dos avanços em técnicas intervencionistas e estratégias de tratamento agudo, o IAMCSST continua sendo uma das principais causas de morbidade e mortalidade em todo o mundo. O advento da FA pós-IAMCSST complica o manejo, levando a desafios persistentes, como alterações hemodinâmicas, disfunção diastólica e aumento do risco de eventos tromboembólicos, incluindo acidente vascular cerebral. Ao elucidar o poder preditivo do NPS, os pesquisadores estão abrindo caminho para a identificação de indivíduos de alto risco que podem se beneficiar de um monitoramento mais intensivo e terapias direcionadas.^6,7^

Um dos aspectos mais importantes do NPS é sua capacidade de sinalizar pacientes com risco aumentado de FANO durante o período crítico após o IAMCSST. A identificação precoce desses indivíduos permite que os médicos implementem medidas proativas, seja por meio de monitoramento mais rigoroso ou do início de estratégias preventivas, para mitigar a incidência de complicações perigosas. As implicações dos resultados do estudo são profundas, pois motivam uma mudança em direção à medicina personalizada, onde as intervenções podem ser adaptadas de acordo com o perfil de risco de cada indivíduo.^1^

Além disso, a metodologia robusta empregada na validação do NPS é louvável. Ao se basear em uma grande coorte, o estudo atinge poder estatístico significativo, aumentando a confiabilidade de suas conclusões. A distinção clara entre pacientes que desenvolvem FANO e aqueles que mantêm ritmo sinusal acrescenta ainda mais clareza à utilidade do NPS em ambientes clínicos.^1^ Esse nível de conhecimento auxilia nos protocolos de tratamento atuais. Abre caminhos para pesquisas futuras, particularmente no que diz respeito à incorporação de marcadores inflamatórios e nutricionais em diversas populações de pacientes, levando, em última análise, a aplicações clínicas mais amplas.

Em uma era declaradamente focada em melhorar os resultados dos pacientes, o potencial do NPS para revolucionar a forma como os médicos abordam os casos de IAMCSST é significativo. Incorporar o NPS à prática clínica de rotina pode gerar uma mudança de paradigma nas abordagens de estratificação de risco e no manejo terapêutico dos pacientes, potencialmente levando a melhores resultados em várias métricas de sucesso.^1^ No entanto, à medida que adotamos esses avanços, a comunidade médica deve agir com cautela, garantindo que ferramentas prognósticas como o NPS sejam utilizadas criteriosamente. A natureza multifatorial da FA deve permanecer na vanguarda da consideração clínica, reconhecendo que, embora os biomarcadores forneçam insights valiosos, eles não levam em conta todos os elementos que influenciam o bem-estar do paciente.

A relação entre inflamação, nutrição e saúde cardiovascular é cada vez mais reconhecida, mas merece maior exploração.^8–10^ O NPS serve como uma base promissora para iniciar esse discurso.^1,3^ Estudos futuros podem expandir os achados iniciais, integrando uma gama mais ampla de biomarcadores e explorando suas interações com comorbidades que frequentemente acompanham o IAMCSST, como diabetes mellitus, insuficiência renal e doença pulmonar obstrutiva crônica.^11^ Essa compreensão holística pode impulsionar inovações em estratégias terapêuticas e permitir que os médicos abordem o estado de saúde abrangente de cada paciente de forma mais eficaz.

Em conclusão, o NPS avança significativamente nossa compreensão dos fatores preditivos associados à FANO no contexto do IAMCSST.^1,3,5^ O NPS fornece uma estrutura inovadora para aprimorar o atendimento ao paciente, entrelaçando os conceitos de inflamação e estado nutricional.^1,3,12^ As implicações dessas descobertas vão além do mero interesse acadêmico; elas têm o potencial de revelar novos insights sobre o manejo de uma das complicações mais complexas e desafiadoras da medicina cardiovascular. Embora a perspectiva otimista em torno do NPS seja justificada, pesquisas contínuas devem ser conduzidas com a máxima cautela e responsabilidade para garantir que os avanços nessa área se traduzam efetivamente em melhores práticas clínicas e desfechos para os pacientes.
