# Supplementary material for: Explorando o Escore Prognóstico de Nápoles: Uma Chave para Prever o Início da Fibrilação Atrial em Casos de IAMCCST
Source: Arq Bras Cardiol. 2025 Jul 8;122(6):e20250218. [Article in Portuguese] doi: 10.36660/abc.20250518 (PMC12269900; doi:10.36660/abc.20250518)
Supplement: Translation

# Translation: Exploring the Naples Prognostic Score: A Key to Predicting New-Onset Atrial Fibrillation in STEMI Cases

**DOI:** 10.36660/abc.20250518i

**Authors:** Paulo Magno Martins Dourado

**Affiliations:** 1 São Paulo SP Brazil Faculdade de Medicina da Universidade de São Paulo, São Paulo, SP – Brazil; 2 São Paulo SP Brazil Clínica Pró-Coração, São Paulo, SP – Brazil

**Keywords:** Atrial Fibrillation, ST Elevation Myocardial Infarction, Prognosis

In the ever-evolving landscape of cardiac care, the Naples Prognostic Score (NPS) has emerged as a promising tool for predicting the onset of atrial fibrillation (AF) in patients experiencing ST-segment elevation myocardial infarction (STEMI).^1^ This innovative scoring system offers insights into patient outcomes and empowers healthcare providers to make real-time, informed decisions. Understanding and utilizing this score can enhance patient management and improve survival rates. Let us explore how the NPS transforms the approach to STEMI and AF prediction.

AF following STEMI is a rising concern in the field of cardiovascular medicine.^2^ The post-MI landscape presents myriad challenges, with AF significantly amplifying the risk of adverse outcomes for patients. A recent study highlighting the NPS brings new hope in the quest to predict and manage complications effectively after myocardial infarction.^3^ Recognizing the potential for early detection of new-onset AF (NOAF) —a condition associated with increased morbidity and mortality—enhances the quality of care delivered to STEMI patients undergoing primary percutaneous coronary intervention.^4^

The NPS is a comprehensive tool that integrates various biomarkers indicative of a pro-inflammatory state, with key components being the neutrophil-to-lymphocyte ratio and the lymphocyte-to-monocyte ratio. These biomarkers are crucial for delineating a patient's inflammatory status and understanding their nutritional status—elements that have become increasingly vital in predicting cardiovascular outcomes. Integrating these parameters allows clinicians to assess risk more effectively, tailored to each patient's unique physiological profile.^5^

The significance of AF following STEMI cannot be overstated. Despite advancements in interventional techniques and acute care strategies, STEMI remains one of the leading causes of morbidity and mortality worldwide. The advent of AF post-STEMI complicates management, leading to persistent challenges such as altered hemodynamics, diastolic dysfunction, and an increased risk of thromboembolic events, including stroke. By illuminating the predictive power of the NPS, researchers are paving the way for identifying high-risk individuals who may benefit from more intensive monitoring and targeted therapies.^6,7^

One of the most potent aspects of the NPS is its capacity to flag patients at an increased risk for NOAF during the critical period following STEMI. Early identification of these individuals enables clinicians to implement proactive measures, be it through closer monitoring or the initiation of preventive strategies, to mitigate the incidence of dangerous complications. The implications of the study's findings are profound, as they motivate a shift toward personalized medicine where interventions can be tailored according to an individual's risk profile.^1^

Moreover, the robust methodology employed in validating the NPS is commendable. By drawing upon a large cohort, the study attains significant statistical power, enhancing the reliability of its conclusions. The clear distinction between patients who develop NOAF and those who maintain sinus rhythm adds further clarity to the utility of the NPS in clinical settings.^1^ This level of insight assists in current treatment protocols. It opens avenues for future research, particularly regarding incorporating inflammatory and nutritional markers across diverse patient populations, ultimately leading to broader clinical applications.

In an era avowedly focused on enhancing patient outcomes, the potential of the NPS to revolutionize how clinicians approach STEMI cases is significant. Incorporating the NPS into routine clinical practice could engender a paradigm shift in risk stratification approaches and the therapeutic management of patients, potentially leading to better outcomes across various success metrics.^1^ However, as we embrace these advancements, the medical community must tread carefully, ensuring that prognostic tools like the NPS are utilized judiciously. The multifactorial nature of AF must remain at the forefront of clinical consideration, acknowledging that while biomarkers provide valuable insights, they do not account for every element influencing patient well-being.

The relationship between inflammation, nutrition, and cardiovascular health is increasingly acknowledged but warrants further exploration.^8–10^ The NPS serves as a promising foundation for beginning that discourse.^1,3^ Future studies could expand upon the initial findings by integrating a wider array of biomarkers and exploring their interactions with comorbidities often accompanying STEMI, such as diabetes mellitus, renal impairment, and chronic obstructive pulmonary disease.^11^ This holistic understanding may drive innovations in therapeutic strategies and enable clinicians to address each patient's comprehensive health status more effectively.

In conclusion, the NPS significantly advances our understanding of predictive factors associated with NOAF within the STEMI context.^1,3,5^ The NPS provides a groundbreaking framework for enhancing patient care by intertwining inflammation and nutritional status concepts.^1,3,12^ The implications of these findings extend beyond mere academic interest; they hold the potential to unlock new insights into managing one of cardiovascular medicine's most complex and challenging complications. While the optimistic outlook surrounding the NPS is warranted, continued inquiry must be conducted with the utmost caution and responsibility to ensure that developments in this area translate effectively into improved clinical practice and patient outcomes.
